# MRPL27 contributes to unfavorable overall survival and disease-free survival from cholangiocarcinoma patients

**DOI:** 10.7150/ijms.50782

**Published:** 2021-01-01

**Authors:** Liping Zhuang, Zhiqiang Meng, Zongguo Yang

**Affiliations:** 1 Department of Integrative Oncology, Fudan University Shanghai Cancer Center, Shanghai 200032, China.; 2 Department of Integrative Medicine, Shanghai Public Health Clinical Center, Fudan University, Shanghai 201508, China.

**Keywords:** Cholangiocarcinoma, MRPL27, Ribosome, Overall survival, Disease-free survival

## Abstract

**Objective**: This study aimed to investigate the roles of MRPL27 in survival from cholangiocarcinoma patients in The Cancer Genome Atlas (TCGA) database.

**Methods**: In TCGA-CHOL profile, MRPL27 gene expression and clinical data were obtained. Cox regression models were used to evaluate the potential links between MRPL27 and cholangiocarcinoma survival. Enrichment analysis of MRPL27 was conducted in Metascape and Gene Set Enrichment Analysis (GSEA) databases.

**Results**: 36 cholangiocarcinoma patients were included in this analysis. MRPL27 mRNA was significantly upregulated in tumor tissues in cholangiocarcinoma patients including intrahepatic, distal and hilar/perihilar cholangiocarcinoma cases (all p < 0.01). Cholangiocarcinoma patients with high MRPL27 had worse overall survival (OS) and disease-free survival (DFS) compared to those with low MRPL27 (all p < 0.05). Univariate and multivariate Cox models indicated that MRPL27 should be a risk factor for the OS and DFS in cholangiocarcinoma patients (both p < 0.01). Bioinformatic analysis revealed that MRPL27 mainly involved in the processes of mitochondrial translation elongation, respiratory electron transport, ATP synthesis, and inner mitochondrial membrane organization. No mutations of MRPL27 were screened in cholangiocarcinoma patients.

**Conclusion**: Upregulated in tumors, MRPL27 contributes to unfavorable survival in cholangiocarcinoma patients.

## Introduction

Cholangiocarcinoma was the most frequent malignancy of the biliary system^1^. Compared to hepatocellular carcinoma, the incidence of cholangiocarcinoma was relatively low^1^, ^2^. However, in United States, the incidence of intrahepatic cholangiocarcinoma (ICC) increased with 4% annually during 1992 to 2000^3^ and doubled from 1976 to 2000^2^, and these similar trends were also reported worldwide^4^, ^5^. Even worse, the prognosis of cholangiocarcinoma is devastating because of its silent clinical character, difficulties in diagnosis and limited therapeutic strategies^6^. The median survival in less than 2 years, the average 5-year survival rate of cholangiocarcinoma is approximately 5% and the mortality of ICC has increased globally^6^-^8^.

Staging systems of cholangiocarcinoma is of great importance for providing information on the prognosis and guidance for therapy. Unfortunately, the majority of the current existed staging systems have proven insufficient for selecting of therapeutic options and lacked prognostic accuracy^6^. Hence, there is an urgent need for reliable prognostic indicators that will enable optimal therapeutic allocation and prognostication. Ribosomes are required for protein production, and are involved in the process of cell proliferation, growth and survival^9^. As member of ribosome pathway, MRPL27 might contribute to the carcinogenesis progression in human cancers including cholangiocarcinoma. However, no study has focused on the oncogenic roles of MRPL27 in cholangiocarcinoma.

In this study, we investigated the expression levels of MRPL27 and correlated it with survival in cholangiocarcinoma patients, in the hope that our findings could be helpful for understanding of pathological process and aggressiveness in cholangiocarcinoma, and useful for identifying novel therapeutic target for cholangiocarcinoma precise treatment.

## Materials and Methods

### Data resource

Patients diagnosed with cholangiocarcinoma from The Cancer Genome Atlas (TCGA) database were included in this study. Transcriptome profiling of MRPL27 with Fragments Per Kilobase per Million (FPKM) standardized were obtained from TCGA (https://portal.gdc.cancer.gov/), and clinical data of cholangiocarcinoma (TCGA, PanCancer Atlas) was downloaded from cBioPortal for cancer genomics (http://www.cbioportal.org/)^10^, ^11^. VLOOKUP index in EXCEL was used to match the gene expression and clinical data. A total of 36 cases were included in this analysis.

### Survival analysis

The survival analysis of MRPL27 for cholangiocarcinoma patients in TCGA-CHOL profile were conducted in Gene Expression Profiling Interactive Analysis web service (GEPIA, http://gepia.cancer-pku.cn/index.html). Outcomes including overall survival (OS) and disease-free survival (DFS) were investigated. Validation analysis of OS and DFS in our matched database was also performed.

### Protein-protein interaction (PPI) and enrichment

PPI of MRPL27 was conducted in Search Tool for Retrieval of Interacting Genes/Proteins (STRING) online service^12^. Interacted genes of MRPL27 were also identified in Search Tool for Interacting Chemicals (STITCH) database^13^. Top 50 similar genes of MRPL27 were identified in GEPIA database^14^.

### Functional Enrichment of interacted genes of MRPL27

Interacted genes and similar genes of MRPL27 in STRING, STITCH and GEPIA were included in the enrichment analysis in Gene Set Enrichment Analysis (GSEA) molecular signatures database^15^, ^16^ and Metascape web service^17^. Top 10 terms of Kyoto Encyclopedia of Genes and Genomes (KEGG) pathway, Gene ontology (GO) biological process and Reactome were described.

### Identification of MRPL27 mutations

Mutations of MRPL27 in cholangiocarcinoma patients were identified in cholangiocarcinoma dataset (TCGA, PanCancer Atlas) from cBioPortal for cancer genomics (http://www.cbioportal.org/)^10^, ^11^. Mutations including missense, truncating, inframe, fusion and others were all screened.

### Statistical analysis

Differences of variables between the individual groups were analyzed using student t test and Mann-Whitney U test based on variables types. Factors associated with the survival were assessed by univariate analysis and multivariate analysis Cox regression. Results were reported as hazard ratios (HR) with 95% confidence intervals (CI). The Kaplan-Meier method with log rank test was used to compare OS and DFS between different groups. Stata software version 16.0 (Stata Corp LLC, Texas, USA) was used. A two-tailed p < 0.05 were considered significant for all tests.

## Results

### MRPL27 expression in cholangiocarcinoma tissues

**Figure 1:**
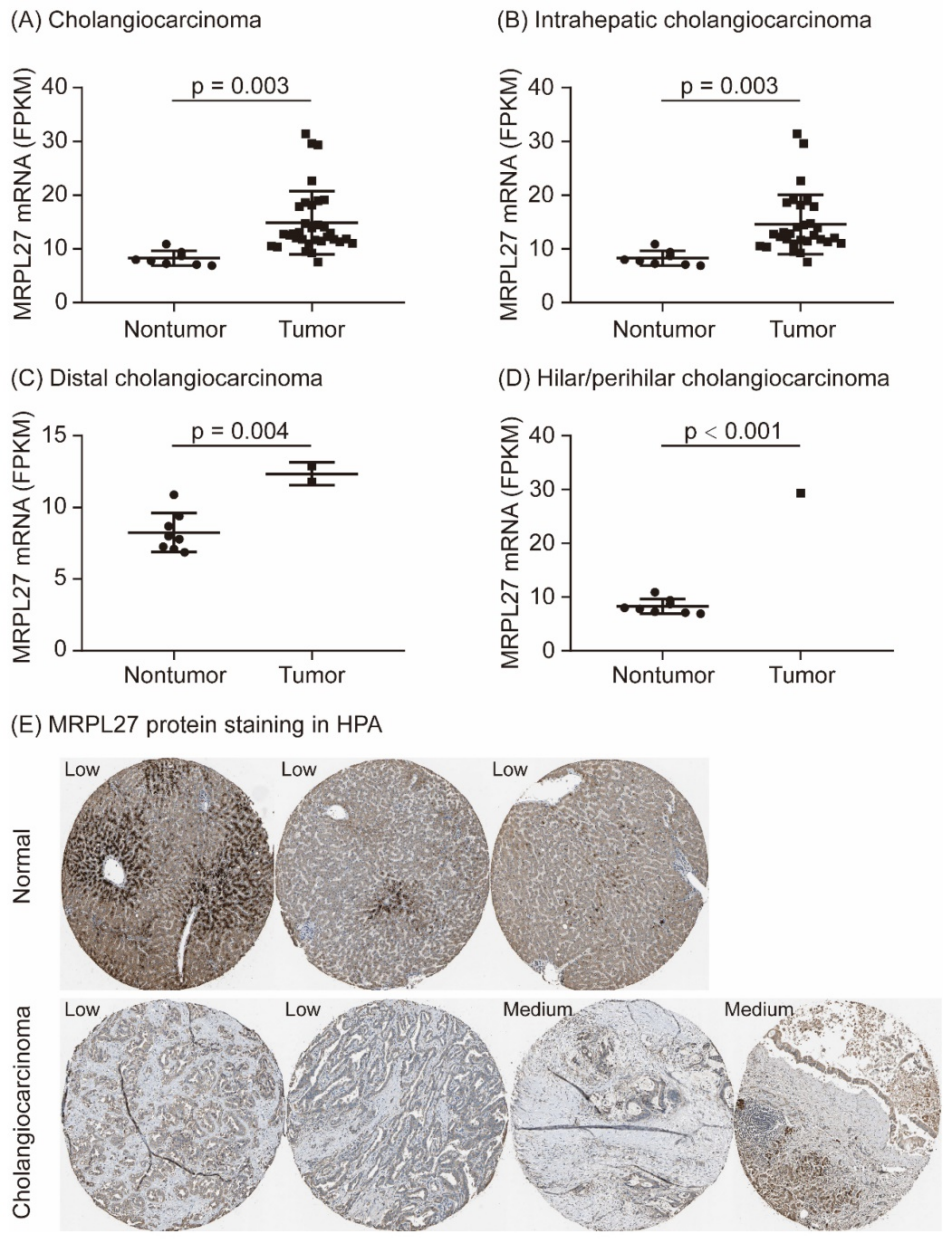
MRPL27 expression in cholangiocarcinoma patients.

As shown in Figure 1, MRPL27 mRNA was significantly upregulated in tumor tissues compared to adjacent normal tissues in cholangiocarcinoma patients (p = 0.003, Figure 1A). According to the tumor locations, MRPL27 mRNA was significantly overexpression in tumor tissues in intrahepatic cholangiocarcinoma, distal cholangiocarcinoma and hilar/perihilar cholangiocarcinoma patients (p = 0.003, p = 0.004 and p < 0.001, respectively, Figure 1B-1D). MRPL27 protein staining was identified in the Human Protein Atlas (https://www.proteinatlas.org/). MRPL27 protein was low stained in three normal liver tissues, while medium stained in two of four cholangiocarcinoma tissues (Figure 1E). In addition, we compared the baseline characteristics of cholangiocarcinoma patients between MRPL27 high and low expression groups. As summarized in Table 1, age, gender, body mass index (BMI), race, family history of cancer, risk factor of cancer, surgical procedure, pathological histology grade, American Joint Committee on Cancer (AJCC) stage, vascular invasion, perineural invasion, fibrosis, new tumor events, alpha-fetoprotein (AFP), total bilirubin, albumin and creatinine levels were equally distributed between the two groups (Table 1).

### Survival analysis of MRPL27 for cholangiocarcinoma patients

**Figure 2:**
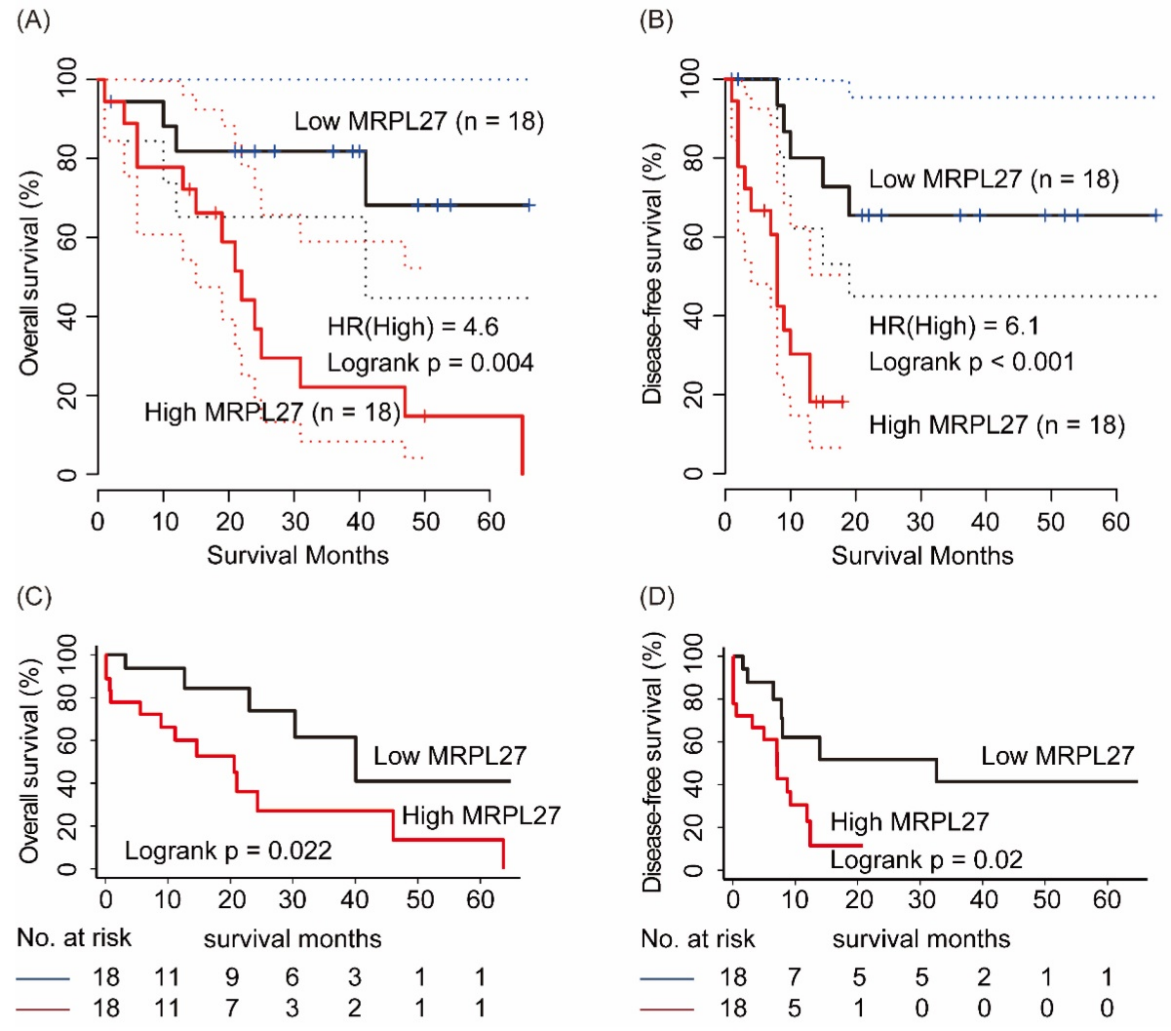
Overall survival and disease-free survival n MRPL27 high and low groups from cholangiocarcinoma patients in GEPIA database (A, B) and in LIHC profile n TCGA (C, D)

In GEPIA online service, 36 cholangiocarcinoma patients were divided into MRPL27 high and low expression groups by median cutoff. As shown in Figure 2, cholangiocarcinoma patients with high MRPL27 in tumor tissues had significantly worse OS and DFS compared to those with low MRPL27 levels (HR = 4.6, p = 0.004 and HR = 6.1, p < 0.001, respectively, Figure 2A and 2B). In validation set, Kaplan-Meier method revealed that high level of MRPL27 in tumor tissues contributed to poorer OS and DFS in cholangiocarcinoma patients (p = 0.022 and p = 0.02, respectively, Figure 2C and 2D).

### Cox models for identification links between MRPL27 and survival in cholangiocarcinoma patients

Variables including MRPL27, age, race, family history of cancer, history of hepatocarcinoma risk factors, surgical procedure, pathological histology grade, AJCC stage, vascular invasion, perineural invasion, ishak scores, new tumor event after original treatment, AFP, total bilirubin, albumin and creatinine were all included in the univariate Cox model. As shown in Table 2, MRPL27, surgical procedure, AJCC stage, vascular invasion, perineural invasion and new tumor event after original treatment might potential factors associated with OS in cholangiocarcinoma patients (p < 0.10). After adjusted these indicators, MRPL27 and vascular invasion were risk factors for OS in cholangiocarcinoma patients in multivariate model (HR = 4.99, p = 0.038 and HR = 11.13, p = 0.007, respectively, Table 2).

As presented in Table 3, MRPL27, surgical procedure, vascular invasion and new tumor event after original treatment were potential indicators for DFS in cholangiocarcinoma patients (p < 0.10). After adjusting surgical procedure, vascular invasion and new tumor event after original treatment, MRPL27 was identified as independent risk factor for DFS in cholangiocarcinoma patients (HR = 5.72, p = 0.013, Table 3).

### Bioinformatic analysis of underlying functions of MRPL27

**Figure 3:**
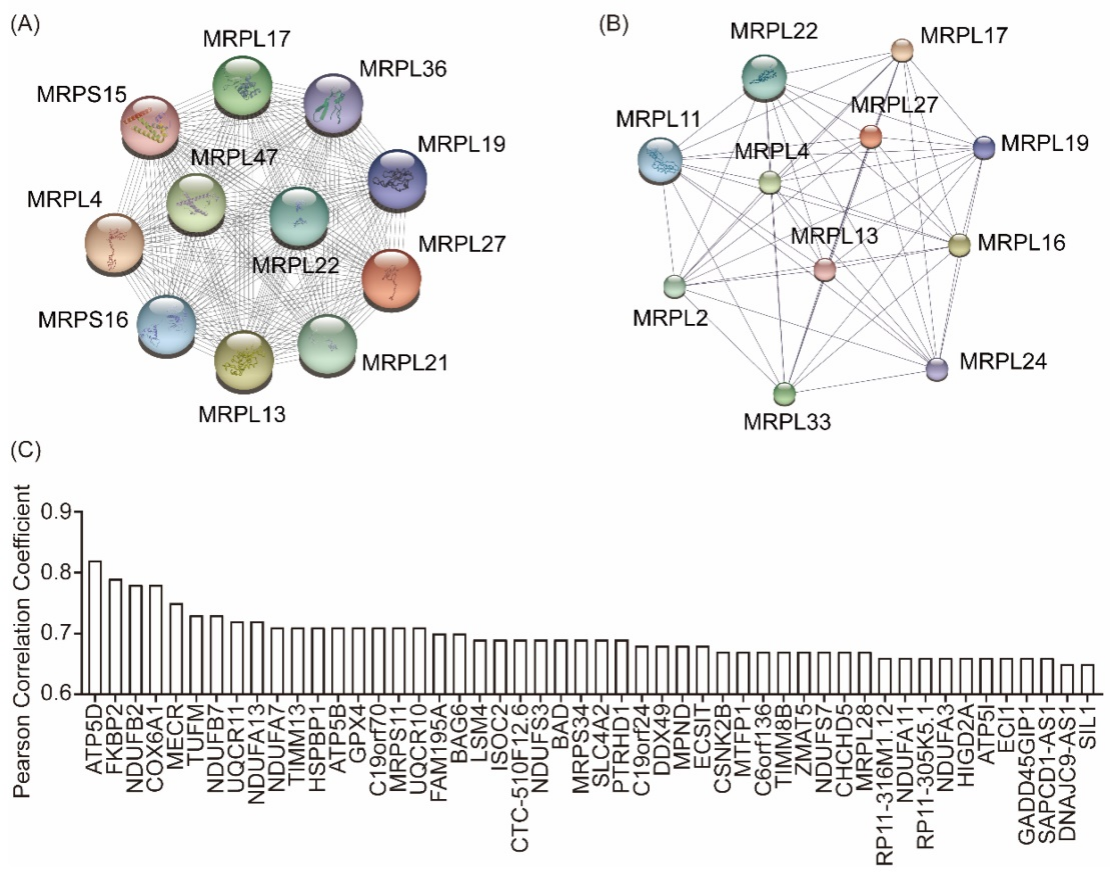
Protein-protein interaction in STRING (A) and STITCH (B), similar genes of MRPL27 in cholangiocarcinoma tumors in GEPIA (C).

To identify the underlying functions of MRPL27 in the development of carcinoma, bioinformatic analysis was performed. PPI analysis in STRING and STITCH indicated that MRPL2, MRPL4, MRPL11, MRPL13, MRPL17, MRPL19, MRPL21, MRPL22, MRPL24, MRPL29, MRPL33, MRPL36, MRPL47, MRPS15 and MRPS16 were interacted with MRPL27 (Figure 3A and 3B). Top 50 similar genes of MRPL27 in GEPIA database were summarized in Figure 3C.

**Figure 4:**
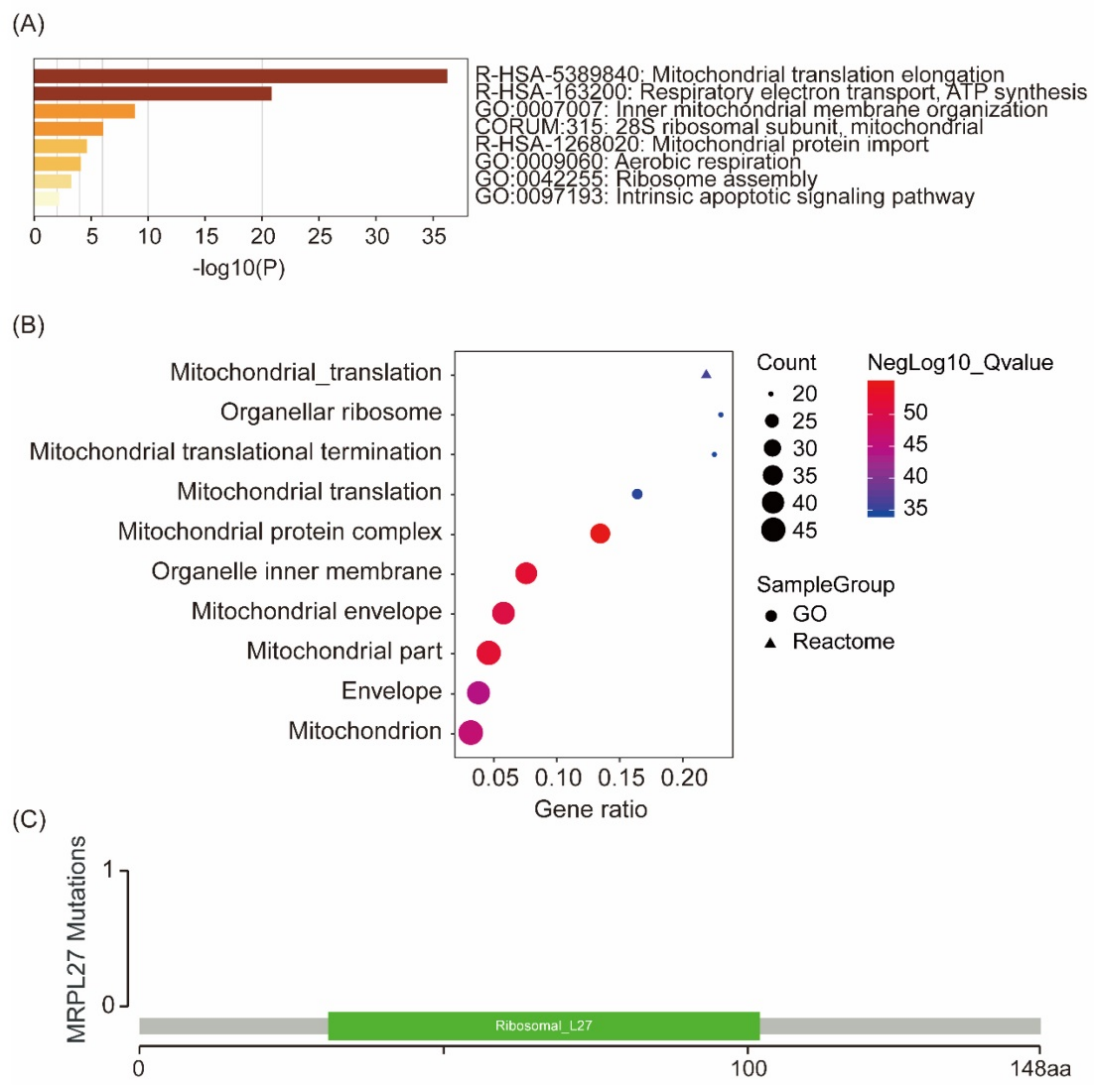
Enrichment of similar genes and interacted genes of MRPL27 in Metascape (A), GSEA (B), and MRPL27 mutation in chilangiocarcinoma from TCGA (C).

Enrichment analysis of interacted and similar genes of MRPL27 showed that MRPL27 mainly involved in the processes of mitochondrial translation elongation, respiratory electron transport, ATP synthesis, and inner mitochondrial membrane organization, which were validated both in Metascape (Figure 4A) and GSEA (Figure 4B) databases. In addition, no mutations of MRPL27 were identified in cholangiocarcinoma patients via screening in cBioPortal for cancer genomics (Figure 4C).

## Discussion

The therapeutic options of cholangiocarcinoma are very limited, resulting in that cholangiocarcinoma has a disappointing prognosis and is almost always irrecoverable owing to its refractory to most currently surgical procedures or medical interventions^18^. Except for tumor locations and pathological subtypes, genetic features also play vital roles in the diagnosis and prognosis in cholangiocarcinoma patients^19^, ^20^. The huge genetic variations remain the main challenge of effective pharmacological therapy^18^. Currently, several molecular pathways including Notch, receptor tyrosine kinase pathways and PI3K-AKT-mTOR pathway, mutations including KRAS, IDH/IDH2, ROS1, FGFR and BAP1 and cytokines including interleukin-6 were identified dysregulated in cholangiocarcinoma^20^.

Ribosome biogenesis serves as one important cellular process that is specially hyperactivated by neoplastic transformation and progression^21^, ^22^. Unfortunately, members in ribosome pathway were not well illustrated in cholangiocarcinoma. Even previous report uncovered that MRPL27 mRNA was downregulated in liver of the metabolic syndrome rats^23^, our analysis indicated that MRPL27 was upregulated in cholangiocarcinoma and associated with unfavorable prognosis including OS and DFS. As a biomarker involved in ribosome production, dyskerin was positive in 56.7% cholangiocarcinoma patients and associated with p53 mutation and a higher proliferative index. Moreover, dyskerin was negatively correlated with DFS in cholangiocarcinoma patients^24^. Located in chromosome X, MRPL27 gene codes for 146 amino acids, YmL27 protein, and involves the large subunit of the mitochondrial ribosome^25^-^27^. In addition, MRPL27 is involved in mitochondrial translation as well as organelle biogenesis and maintenance^28^, in which in line with our results from bioinformatic analysis. Gruschke S et al revealed a complex network of interacting proteins including MBA1, MRPL3, MRPL13 as well as MRPL27 specific to mitochondrial ribosomes. And these proteins constitute ribosomal proteins exclusively found in mitochondria and promote oxidative phosphorylation in mitochondria^29^. Our bioinformatic analysis also indicated that MRPL27 contributed to ATP synthesis, which together with oxidative phosphorylation might involve in the tumor energy support system and contribute to cancer aggressiveness^30^-^32^. Additionally, no mutations of MRPL27 were screened in cholangiocarcinoma patients in our research. Taken together, we speculated that MRPL27 might be a potential candidate target for cancer therapy including cholangiocarcinoma.

Indeed, our study has some limitations. Firstly, this analysis included small samples of cholangiocarcinoma patients, leading to relatively low representative of this population. In our study, most of cholangiocarcinoma cases were intrahepatic cholangiocarcinoma, while the sample counts of distal cholangiocarcinoma and hilar/perihilar cholangiocarcinoma were low. Hence, the accuracy and reliability of these calculation results should be guaranteed in prospective studies with large samples. Secondly, we could not conduct experimental research for probing potential oncogenic mechanisms of MRPL27 in cholangiocarcinoma development. Thirdly, this analysis in a bioinformatic study based on TCGA dataset, there was no follow-up data for our own from available cholangiocarcinoma patients.

According to our preliminary findings, we cautiously concluded that upregulation of MRPL27 in tumor tissues predicted worse OS and DFS in cholangiocarcinoma patients. MRPL27 mainly involved in the processes of mitochondrial translation elongation, respiratory electron transport, ATP synthesis, and inner mitochondrial membrane organization.

## Figures and Tables

**Table 1:** Baseline characteristics of cholangiocarcinoma patients in MRPL27 high and low expression groups

Variables	MRPL27 high group (n = 18)	MRPL27 low group (n = 18)	p value
Age, years, mean ± SD	64.1 ± 2.5	61.9 ± 3.5	0.62
Male, n (%)	8 (44.4)	8 (44.4)	1.0
Body mass index (BMI), n (%)			0.251
BMI < 19.9	2 (11.1)	0 (0)	
20 ≤ BMI < 24.9	5 (27.8)	3 (16.7)	
25 ≤ BMI < 29.9	8 (44.4)	8 (44.4)	
BMI ≥ 30	3 (16.7)	7 (38.9)	
White race, n (%)	16 (88.9)	15 (83.3)	0.63
Family history of cancer, n (%)	10 (55.6)	11 (61.1)	0.735
Risk factor of cancer, n (%)			0.735
No	7 (38.9)	8 (44.4)	
Yes	11 (61.1)	10 (55.6)	
Surgical therapy, n (%)			0.239
Other, Specify	2 (11.1)	0 (0)	
Segmentectomy, Single	2 (11.1)	1 (5.6)	
Pancreaticoduodenectomy, Whipple Resection	5 (27.8)	9 (50.0)	
Segmentectomy, Multiple	3 (16.7)	0 (0)	
Extended Lobectomy	3 (16.7)	4 (22.2)	
Lobectomy	3 (16.7)	4 (22.2)	
Grade, n (%)			0.502
I-II	9 (50.0)	11 (61.1)	
III-IV	9 (50.0)	7 (38.9)	
AJCC stage, n (%)			0.718
I	9 (50.0)	10 (55.6)	
II	4 (22.2)	5 (27.8)	
III-IV	5 (27.8)	3 (16.7)	
Vascular invasion, n (%)	3 (16.7)	4 (22.2)	0.674
Perineural invasion, n (%)	5 (27.8)	2 (11.1)	0.206
Fibrosis (Ishak score), n (%)	6 (33.3)	5 (27.8)	0.717
New tumor event after original treatment, n (%)	10 (55.6)	7 (38.9)	0.317
Alpha-fetoprotein (AFP), mean ± SD	4.02 ± 0.91	3.44 ± 0.53	0.6
Total bilirubin, mean ± SD	0.72 ± 0.13	0.64 ± 0.07	0.585
Albumin, mean ± SD	4.13 ± 0.12	4.04 ± 0.14	0.652
Creatinine, mean ± SD	0.85 ± 0.06	0.87 ± 0.04	0.732

**Table 2:** Parameters associated with OS in cholangiocarcinoma patients in TCGA database^#^

Variables	Univariate				Multivariate		
	HR	95%CI	P value		HR	95%CI	P value
MRPL27, median							
Low	Reference	-	1.0		Reference	-	1.0
High	3.15	1.12 - 8.89	0.03		4.99	1.09 - 22.82	**0.038**
Surgical procedure							
Other, Specify	Reference	-	1.0		Reference	-	1.0
Segmentectomy, Single	0.53	0.07 - 3.95	0.537		0.10	0.01 - 1.30	0.078
Pancreaticoduodenectomy, Whipple Resection	0.10	0.02 - 0.59	0.011		0.11	0.01 - 1.12	0.063
Segmentectomy, Multiple	0.17	0.01 - 1.92	0.152		0.11	0 - 3.38	0.209
Extended Lobectomy	0.07	0.01 - 0.50	0.009		0.08	0.01 - 1.16	0.065
Lobectomy	0.18	0.03 - 1.22	0.078		0.24	0.02 - 2.30	0.214
AJCC stage							
I	Reference	-	1.0		Reference	-	1.0
II	2.98	0.91 - 9.76	0.072		6.36	1.0 - 40.53	0.05
III-IV	1.78	0.56 - 5.64	0.325		1.39	0.28 - 7.05	0.689
Vascular invasion							
No	Reference	-	1.0		Reference	-	1.0
Yes	3.15	1.03 - 9.66	0.045		11.13	1.92 - 64.45	**0.007**
Perineural invasion							
No	Reference	-	1.0		Reference	-	1.0
Yes	3.56	0.97 - 13.07	0.056		1.74	0.25 - 12.07	0.576
New tumor event after original treatment							
No	Reference	-	1.0		Reference	-	1.0
Yes	2.61	0.85 - 8.01	0.094		1.70	0.26 - 11.11	0.581

Variables including MRPL27, age, race, family history of cancer, history of hepatocarcinoma risk factors, surgical procedure, pathological histology grade, AJCC stage, vascular invasion, perineural invasion, ishak scores, new tumor event after original treatment, AFP, total bilirubin, albumin and creatinine were all included in the univariate Cox model. ^#^Only variables significantly associated with OS in univariate analysis (p < 0.10) were presented.

**Table 3:** Parameters associated with DFS in cholangiocarcinoma patients in TCGA database^#^

Variables	Univariate				Multivariate		
	HR	95%CI	P value		HR	95%CI	P value
MRPL27, median							
Low	Reference	-	1.0		Reference	-	1.0
High	3.02	1.13 - 8.06	0.027		5.72	1.45 - 22.60	**0.013**
Surgical procedure							
Other, Specify	Reference	-	1.0		Reference	-	1.0
Segmentectomy, Single	0.40	0.05 - 2.91	0.365		0.34	0.04 - 2.57	0.294
Pancreaticoduodenectomy, Whipple Resection	0.26	0.05 - 1.28	0.098		0.29	0.05 - 1.74	0.175
Segmentectomy, Multiple	0.11	0.01 - 1.35	0.085		0.14	0.01 - 2.62	0.189
Extended Lobectomy	0.15	0.02 - 0.95	0.044		0.21	0.03 - 1.49	0.119
Lobectomy	0.30	0.05 - 1.76	0.183		0.38	0.06 - 2.60	0.326
Vascular invasion							
No	Reference	-	1.0		Reference	-	1.0
Yes	5.11	1.76 - 14.87	0.003		13.14	2.97 - 58.10	**0.001**
New tumor event after original treatment							
No	Reference	-	1.0		Reference	-	1.0
Yes	5.20	1.71 - 15.74	0.004		3.20	0.79 - 13.0	0.103

Variables including MRPL27, age, race, family history of cancer, history of hepatocarcinoma risk factors, surgical procedure, pathological histology grade, AJCC stage, vascular invasion, perineural invasion, ishak scores, new tumor event after original treatment, AFP, total bilirubin, albumin and creatinine were all included in the univariate Cox model. ^#^ Only variables significantly associated with DFS in univariate analysis (p < 0.10) were presented.
